# Glia in FTLD-*GRN*: from supporting cast to leading role

**DOI:** 10.1172/JCI168215

**Published:** 2023-03-15

**Authors:** Emile S. Pinarbasi, Sami J. Barmada

**Affiliations:** 1 Department of Pathology and; 2 Department of Neurology, University of Michigan, Ann Arbor, Michigan, USA.

## Abstract

A subset of the neurodegenerative disease frontotemporal lobar degeneration (FTLD) is caused by mutations in the progranulin (*GRN*) gene. In this issue of the *JCI*, Marsan and colleagues demonstrate disease-specific transcriptional profiles in multiple glial cell lineages — astrocytes, microglia, and oligodendroglia — that are highly conserved between patients with FTLD-*GRN* and the widely used *Grn^–/–^* mouse model. Additionally, the authors show that *Grn^–/–^* astrocytes fail to adequately maintain synapses in both mouse and human models. This study presents a compelling argument for a central role for glia in neurodegeneration and creates a rich resource for extending mechanistic insight into pathophysiology, identifying potential biomarkers, and developing therapeutic approaches.

## The missing link between progranulin and TDP-43

Neurodegenerative diseases, with their relentlessly progressive clinical course and sparse therapeutic options, are responsible for substantial morbidity and mortality worldwide. These disorders are characterized clinically by progressive motor and/or cognitive dysfunction and pathologically by the accumulation of misfolded proteins in vulnerable neuronal populations. Most genetic mutations associated with neurodegenerative conditions directly affect the production, solubility, intracellular localization, or turnover of aggregate-prone proteins. However, FTLD-*GRN* is a notable exception. In this subset of frontotemporal lobar degeneration with TDP-43 inclusions (FTLD-TDP), patients have a heterozygous loss-of-function mutation in *GRN*, resulting in haploinsufficiency of the secreted protein progranulin. The mechanism by which progranulin haploinsufficiency leads to neurodegeneration and the characteristic TDP-43 protein aggregates remains an important yet unanswered question.

Progranulin is a secreted, cysteine-rich protein that is proteolytically cleaved into seven granulin proteins with potential functions in lysosomal degradation and neuroinflammation. In contrast to the late-onset neurodegenerative disease associated with heterozygous *GRN* mutations, homozygous loss-of-function *GRN* mutations result in neuronal ceroid lipfuscinosis, a pediatric lysosomal storage disease appearing with seizures, developmental delay, and vision loss (1). Consistently, progranulin regulates lysosomal acidification and function in cultured cells, and there is evidence of lysosomal dysfunction in *Grn* KO (*Grn*^–/–^) mice as well as in the brains of patients with FTLD-*GRN* (2, 3). In addition, *Grn*^–/–^ mice demonstrate increased inflammation in response to a variety of insults including infection, toxin exposure, and traumatic brain injury (4–6). Progranulin is also essential for microglial lipid metabolism; this function is particularly pertinent given the appearance of lipid droplets in a proinflammatory subset of microglia found in aging mouse and human brains (7).

## Glia move from backdrop to center stage in FTLD-*GRN*

**Figure 1:**
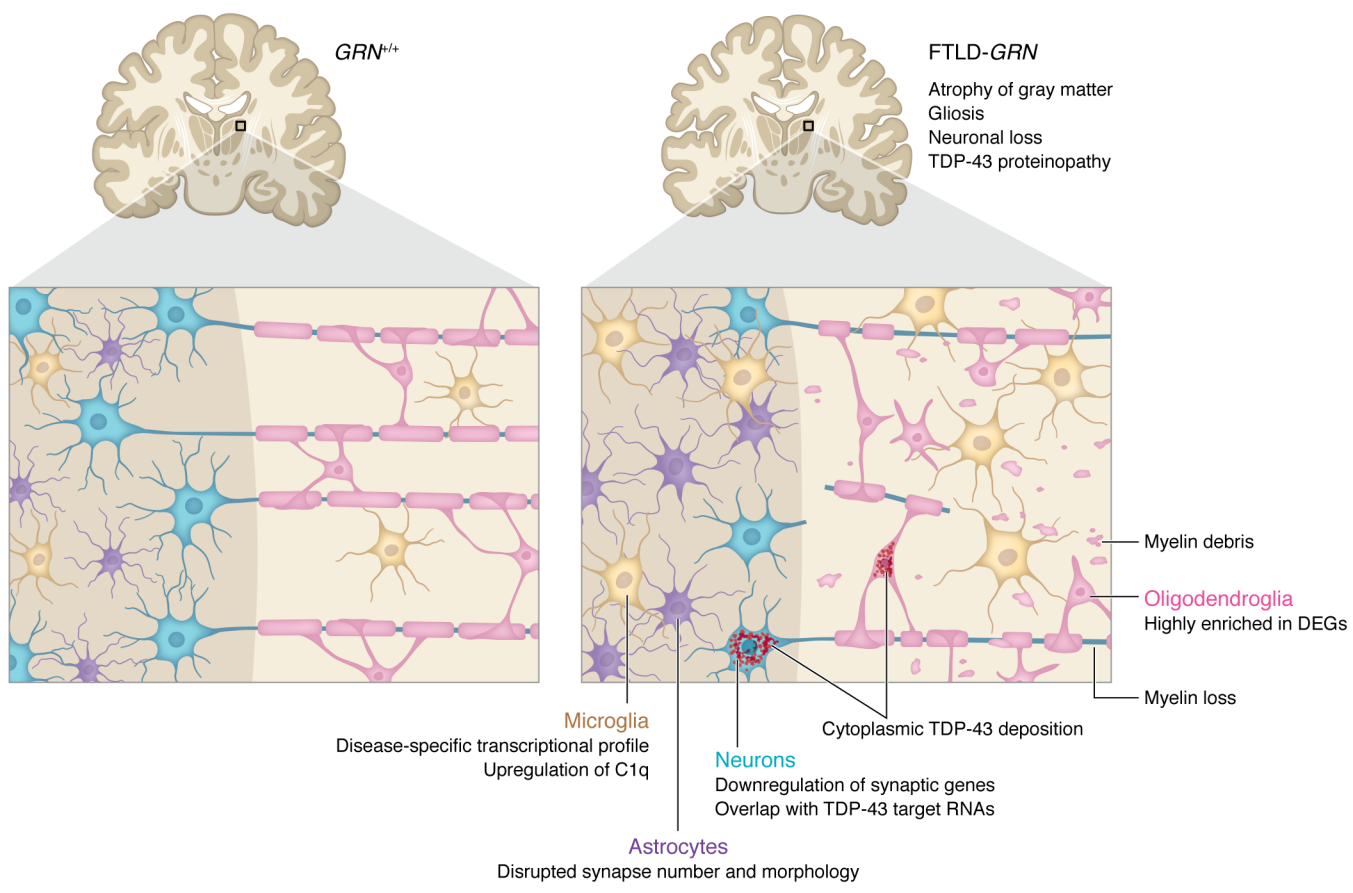
Noncell autonomous mechanisms contribute to FTLD-*GRN*. Multiple cell and tissue types factor into neurodegeneration in FTLD-*GRN*. Data from Marsan et al. (10) indicate that *GRN* deficiency induces downstream changes in all four cell types of the brain. Atrophy, gliosis, neuronal loss, and TDP-43 poteinopathy are prominent in gray matter, evident at both the subcellular and transcriptional levels. Microglia increase synaptic pruning and display a disease-specific transcriptional profile, including the upregulation of C1q. Astrocytes fail to maintain synapses, resulting in disrupted synapse number and morphology. The downregulation of synaptic genes in neurons include TDP-43 target RNAs associated with TDP-43 nuclear exclusion and cytoplasmic deposition. Although TDP-43 proteinopathy is mild and variable in white matter, other pathological changes, including gliosis, myelin loss, and myelin debris within microglia, are more common. DEGs are highly enriched in oligodendroglia, suggesting that these cells are substantially impacted in FTLD-*GRN*.

While hemizygous *Grn* (*Grn*^+/–^) mice display only mild behavioral phenotypes and no neuropathologic abnormalities, *Grn*^–/–^ mice recapitulate key clinical and neuropathologic features of FTLD. These animals display age-associated learning and memory deficits together with TDP-43 aggregates, neuronal loss, and gliosis in the thalamus and hippocampus (2, 4, 8, 9). In this issue of the *JCI*, Marsan, et al. use NanoString, a single nucleus RNA sequencing (snRNA-Seq) technique, to uncover cell-type specific transcriptional changes in FTLD-*GRN* human and *Grn*^–/–^ mouse brains (10). In so doing, they present a compelling argument for neuroinflammation and the noncell autonomous contributions of glia to neurodegeneration in FTLD-*GRN* (Figure 1). Moreover, their comprehensive study provides a thorough characterization of *Grn*^–/–^ mice, highlighting where this common model of FTLD accurately mimics human disease, while also revealing important discrepancies.

Microglia, the resident inflammatory cells of the brain, are increasingly recognized for their contribution to neurodegeneration and disease progression. Previous snRNA-Seq studies of microglia in *Grn*^–/–^ mice demonstrated a mutant-specific transcriptional profile, similar to disease-activated microglia (DAM) profiles seen in Alzheimer’s disease and amyotrophic lateral sclerosis (ALS) (8). Marsan and colleagues build upon this work by (a) expanding the cell types assessed to include neurons, astrocytes, and other glial cells; (b) complementing the studies in *Grn*^–/–^ mice with snRNA-Seq of postmortem patient’s brain tissue with FTLD-*GRN*; and (c) independently evaluating the cortex and thalamus from both mouse and human samples (10).

Importantly, these experiments showed a considerable overlap in microglial differentially expressed genes (DEGs) from each model, particularly within the thalamus. Moreover, unsupervised trajectory and pseudotime analyses of thalamic microglia emphasized the relevance of these transcriptional changes to disease and prognosis. Two trajectories were highly correlated with FTLD-*GRN*, one of which predicted shorter disease duration. Among the microglial DEGs associated with FTLD-*GRN* in humans and *Grn* loss in mice, C1q stands out. *Grn*^–/–^ microglia actively prune the synapses of cocultured neurons in a C1q-dependent manner (11), and genetic deletion of C1q in *Grn*^–/–^ mice partially ameliorates neurodegeneration within the thalamus (11). Notably, prior work suggests that cultured media from *Grn*-deficient microglia — presumably rich in proinflammatory, secreted molecules such as C1q — induces cytoplasmic accumulation of TDP-43 in cultured neurons (8).

Marsan, et al. also unearthed an unanticipated contribution from astrocytes to neurodegeneration in FTLD-*GRN*. Specifically, *Grn*^–/–^ astrocytes failed to adequately maintain synapses in both mouse and human models, and, in fact, actively reduced synapse number and disrupted synapse morphology. While conditioned media from *Grn*^+/+^ astrocytes enhanced synapse number in both WT and mutant neurons, *Grn*^–/–^ astrocyte conditioned media had the opposite effect. To pursue this phenomenon in a human model system, the authors differentiated astrocytes from induced pluripotent stem cells (iPSCs) before engrafting them into cortical organoids. Unlike in mice, the number of synapses was unaffected by *GRN*^–/–^ astrocytes. However, organoids engrafted with *GRN*^–/–^ astrocytes exhibited abnormally large synapses that appeared morphologically similar to those in cortical organoids without astrocytes (10). While this result implied that *GRN*^–/–^ astrocytes were impeding synapse function and/or maturation, further studies are needed to confirm the functional readout of this morphologic change.

As focus has shifted to the contributions of glial cells to neurodegeneration, evidence of a role for oligodendroglia has emerged (12). Marsan and colleagues also uncovered a striking enrichment for oligodendroglial DEGs in the human FTLD-*GRN* thalamus, implying that oligodendrocytes may be substantially affected in disease (10). FTLD-*GRN* is marked by prominent white matter hyperintensities on brain MRIs, the severity of which correlate with disease progression (13). These hyperintensities indicate areas of gliosis and myelin loss (14, 15) that are also seen in *Grn*^–/–^ mice. Furthermore, myelin debris accumulate within the lysosomes of white matter microglia in tissue from patients with FTLD-*GRN* and *Grn*^–/–^ mice (9), and proteomic studies revealed reduced oligodendrocyte and myelin markers in *Grn*^–/–^ animals (9, 16). Together, these observations add FTLD-*GRN* to the growing list of neurodegenerative disorders linked with oligodendroglial dysfunction and white matter disease, albeit through unknown mechanisms.

The mislocalization and cytoplasmic deposition of TDP-43, which is essential for oligodendrocyte maturation, myelination, and survival, offers a potential explanation for white matter pathology in patients with FTLD-*GRN* (17, 18). However, TDP-43 inclusions in FTLD-*GRN* rarely appear in oligodendroglia and are instead concentrated within neurons, and, less so, in microglia (19). As such, the link between progranulin, TDP-43, and white matter pathology in FTLD-*GRN* is unclear but alluring.

## Evidence of TDP-43 loss of function

TDP-43 is a highly conserved RNA-binding protein with important roles in RNA splicing, stability, and transport (20, 21). TDP-43 is typically concentrated within the nucleus, but in FTLD-TDP and FTLD-*GRN*, it is excluded from the nucleus and accumulates within cytoplasmic inclusions (22). Neuronal dysfunction and death may ensue from the loss of crucial TDP-43 splicing activity in the nucleus, toxicity associated with its cytoplasmic deposition, or a combination thereof. The origins of TDP-43 mislocalization, and whether it can be reversed, remain unknown.

Evidence of TDP-43 loss of function — as judged by the increase in unannotated or cryptic splicing events within susceptible transcripts, such as stathmin-2 (STMN2) — has emerged from studies of FTLD-TDP (23) but has yet to be explored in FTLD-*GRN*. To pursue this question, Marsan et al. cross referenced neuronal DEGs detected in FTLD-*GRN* samples with transcripts previously determined to be misspliced in a TDP-43-dependent manner from FTLD-TDP (24, 25) or iPSC-derived neurons (26). They observed notable overlap in human thalamus, human frontal cortex, and mouse thalamus (all of which exhibit TDP-43 pathology), but only minimal overlap in the mouse frontal cortex (which does not develop TDP-43 pathology). A caveat to this approach is the clear differences in TDP-43 substrates and splicing targets between mouse and humans (20). In addition, gene expression analysis with the Nanostring platform does not allow for assessment of distinct splice isoforms, preventing accurate distinction of splicing events that may indicate TDP-43 loss-of-function in human or mouse samples. Even so, Marsan and colleagues provide the first hint of specific TDP-43 loss-of-function in FTLD-*GRN* tissue and *Grn*^–/–^ mice, as would be expected based on TDP-43 pathology.

## The unique niche of frontal cortex

While *Grn*^–/–^ mice in many ways accurately model FTLD-*GRN*, they do not fully recapitulate the pathology that is so closely tied to the cognitive and behavioral symptoms of FTLD. *Grn*^–/–^ mice exhibit relatively mild gliosis and microglial infiltration of cortex and lack the often dramatic, cortical atrophy seen in human disease. The cortex of *Grn*^–/–^ mice also shows little TDP-43 aggregation or mislocalization. In vitro cultures of primary neurons from these animals demonstrate TDP-43 mislocalization, however, implying that TDP-43 pathology may emerge under specific conditions (8).

Studies performed by Marsan et al. confirm this discrepancy (10). Mouse frontal cortex displayed the least overlap between human frontal cortex and thalamus when comparing single cell transcriptomics of microglia, astrocytes, and neurons. Furthermore, unsupervised pseudotime and trajectory analyses of frontal cortex microglia failed to yield disease-specific trajectories, unlike thalamic microglia (10). One possible explanation for these findings is a unique, species-specific repertoire of TDP-43 dependent splicing events, leading to distinct consequences for TDP-43 pathology in the mouse frontal cortex compared with the same region in humans. Alternatively, subpopulations of susceptible cells in FTLD, such as Von Economo neurons (27), may be preferentially abundant in humans compared with rodents.

## Conclusion and clinical implications

The most striking neuropathologic features of neurodegenerative diseases are protein aggregates. Understandably, this pathology has led to the hypothesis that aggregates are inevitably tied to, and directly responsible for, neurodegeneration in these conditions. Current therapeutic strategies aimed at reducing aggregates or aggregate-prone proteins have produced underwhelming results in clinical trials (28) or are far from ready for testing in humans (29). For these reasons among others, focus has shifted toward neuroinflammation and immune modulation for therapeutic purposes in FTLD and related conditions. Through a comprehensive and systematic analysis of single cell transcriptomics, Marsan and colleagues expose fundamental mechanisms by which glia — astrocytes, microglia, and oligodendrocytes alike — contribute to neurodegeneration in FTLD-*GRN*. In so doing, they have created a rich resource and a strong basis for extending insight into disease pathophysiology, identifying potential biomarkers, and developing alternative approaches to therapy in FTLD.

## Version 1 03/15/2023

Electronic publication
